# A System for Computational Assessment of Hand Hygiene Techniques

**DOI:** 10.1007/s10916-022-01817-z

**Published:** 2022-05-06

**Authors:** Chaofan Wang, Weiwei Jiang, Kangning Yang, Zhanna Sarsenbayeva, Benjamin Tag, Tilman Dingler, Jorge Goncalves, Vassilis Kostakos

**Affiliations:** School of Computing and Information Systems, The University of Melbourne, 700 Swanston St, Carlton, 3053 VIC Australia

**Keywords:** hand hygiene, handrub, six-step hand hygiene technique, healthcare-associated infections, nosocomial infections, 68-00, 68U07, 68U10

## Abstract

The World Health Organization (WHO) recommends a six-step hand hygiene technique. Although multiple studies have reported that this technique yields inadequate skin coverage outcomes, they have relied on manual labeling that provided low-resolution estimations of skin coverage outcomes. We have developed a computational system to precisely quantify hand hygiene outcomes and provide high-resolution skin coverage visualizations, thereby improving hygiene techniques. We identified frequently untreated areas located at the dorsal side of the hands around the abductor digiti minimi and the first dorsal interosseous. We also estimated that excluding Steps 3, 6R, and 6L from the six-step hand hygiene technique leads to cumulative coverage loss of less than 1%, indicating the potential redundancy of these steps. Our study demonstrates that the six-step hand hygiene technique could be improved to reduce the untreated areas and remove potentially redundant steps. Furthermore, our system can be used to computationally validate new proposed techniques, and help optimise hand hygiene procedures.

## Introduction

Healthcare-Associated Infections (HAIs) or nosocomial infections are a major patient-safety challenge in healthcare settings [1]. Appropriate hand hygiene is a simple and cost-efficient measure to avoid the transmission of pathogens and prevent HAIs [1].

**Fig. 1:**
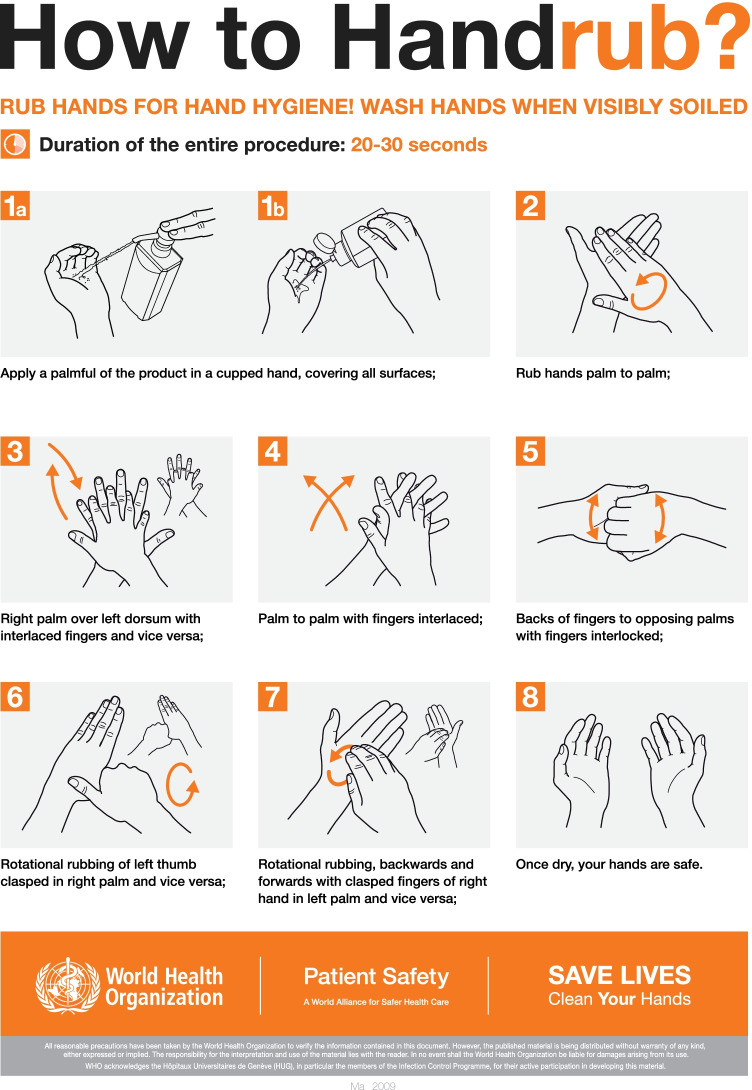
Standard World Health Organization procedures of alcohol-based handrub [1]

However, research has found that hand hygiene quality in healthcare settings is generally unsatisfactory [2, 3]. Taylor evaluated Healthcare Workers (HCWs)’ handwash procedures and observed that their procedures often miss parts of the hands, indicating low effectiveness of individual handwash techniques [2, 3]. Based on the summarized untreated areas, Ayliffe et al. proposed a 30-second six-step hand hygiene technique for both handwash and handrub to ensure that the hands are completely covered with hand disinfectants [4]. Nowadays, the proposed six-step hand hygiene technique has been adopted in the EN 1500 standard [5] and the “World Health Organization (WHO) guidelines on Hand Hygiene in Health Care” [1] (Fig. 1). Yet, research has shown that the resulting skin coverage when using this technique is often inadequate [6, 7]. Others have proposed to modify the six-step hand hygiene technique by introducing additional steps to cover the untreated areas [6–8]. In addition, research has also reported that HCWs’ adherence to all steps of the six-step hand hygiene technique is low [8, 9]. Thus, several simplified alternatives to the six-step hand hygiene technique have been proposed to increase HCWs’ compliance and adherence [7, 8].

The effectiveness of a proposed hand hygiene technique may be assessed by two methods: microbiological validation and Ultraviolet (UV) tests. Microbiological validation mainly uses samples from the fingertips (EN 1500) [5] or through the glove juice method (ASTM E-1174) before and after hand hygiene procedures; thereby, hand hygiene quality is evaluated in terms of bacteria count reduction [1]. Conversely, UV tests require that test subjects use a hand disinfectant mixed with fluorescent concentrates to perform the handrub procedure, and then measure the skin coverage of the fluorescent handrub [7]. Compared to microbiological validation, UV tests can deliver an immediate and well-visible result of skin coverage with hand disinfectants [10]. Furthermore, a strong correlation between the visual evaluation of UV tests and the degree of bacterial count reduction has been reported [11, 12].

However, a drawback of previous studies using UV tests is that data aggregation from multiple participants was either coarse or manual, leading to low-resolution assessments and sacrificing an opportunity to generate high-resolution granular insights on the technique’s effectiveness.

In this paper, we present a system to computationally assess and combine the resulting images from multiple UV tests to document the untreated areas using standard hand templates. We evaluated the system in a lab study, where we measured skin coverage after performing the complete six-step hand hygiene technique and its individual steps. By aggregating the computed hand templates, we precisely identified the frequently untreated areas. We also computationally estimated the coverage loss when excluding individual steps from the technique, thus identifying potentially redundant steps.

## Method

### Study Design and Participants

This study was designed to computationally evaluate the effectiveness of the six-step hand hygiene technique through UV tests. The study was conducted at the University of Melbourne, Australia. The study protocol was reviewed and approved by the University of Melbourne’s Human Ethics Advisory Group.

Participants were recruited through the university’s mailing lists and snowball recruitment with an equal number of women and men. Participants were excluded if they had a history of allergic reactions to alcohol handrub and UV light. All participants were students or staff members at our university, and their ages ranged from 18 to 27 (M = 22.8, SD = 2.1 years). Non-HCWs were chosen to have participants who were not routinely acquainted with the six-step hand hygiene technique to allow a better assessment of its practicality and effectiveness [7].

### Experiment Procedures

Upon arrival at our lab, we briefed the participants on the study purpose and obtained their written consent to participate in the experiment. We subsequently provided training to our participants on how to perform the six-step technique by first explaining the steps presented in Fig. 1. They then watched an instructional video provided by the WHO three times [13]. While watching, they were asked to perform the six-step technique for training.

After the training session, participants proceeded to complete the experimental tasks. We designed two types of tasks where participants used fluorescent handrub to cover their hands. The first type of task (individual step) was to perform either Step 1 alone or Step 1 followed by each individual step from the six-step hand hygiene technique described in Table 1 separately, using 3 ml alcohol handrub [1]. Step 1 was a necessary prerequisite for other steps, because only Step 1 allows spreading alcohol handrub that further enables performing the remaining steps. The second type of task (complete technique) was to perform the complete six-step technique using 3 ml alcohol handrub [1]. Details of each task have been described in Table 2.

**Table 1:** Step description of the six-step hand hygiene technique

Step#	Description of individual step
1	Rub hands palm to palm
2R	Right palm over left dorsum with interlaced fingers
2L	Left palm over right dorsum with interlaced fingers
3	Palm to palm with fingers interlaced
4	Backs of fingers to opposing palms with fingers interlocked
5R	Rotational rubbing of left thumb clasped in right palm
5L	Rotational rubbing of right thumb clasped in left palm
6R	Rotational rubbing, backwards and forwards with clasped fingers of right hand in left palm
6L	Rotational rubbing, backwards and forwards with clasped fingers of left hand in right palm

R: right hand; L: left hand.

**Table 2:** description for the experiment

**Experimental task**	**Hand side**	**Task type**	**Step**
1	Dorsal	Individual step	1
2	Dorsal	Individual step	1 + 2R
3	Dorsal	Individual step	1 + 2L
4	Dorsal	Individual step	1 + 3
5	Dorsal	Individual step	1 + 4
6	Dorsal	Individual step	1 + 5R
7	Dorsal	Individual step	1 + 5L
8	Dorsal	Individual step	1 + 6R
9	Dorsal	Individual step	1 + 6L
10	Dorsal	Complete technique	-
11	Dorsal	Complete technique	-
12	Palmar	Individual step	1
13	Palmar	Individual step	1 + 2R
14	Palmar	Individual step	1 + 2L
15	Palmar	Individual step	1 + 3
16	Palmar	Individual step	1 + 4
17	Palmar	Individual step	1 + 5R
18	Palmar	Individual step	1 + 5L
19	Palmar	Individual step	1 + 6R
20	Palmar	Individual step	1 + 6L
21	Palmar	Complete technique	-
22	Palmar	Complete technique	-

R: right hand; L: left hand

To minimize the potential for participant fatigue and skin irritation, we limited the experiment duration by only observing one side of each participants’ hands (either palmar side or dorsal side) for the tasks of individual steps. Specifically, all participants completed tasks 10, 11, 21, and 22 (two repeated tasks of complete technique for both sides). Additionally, half of the cohort completed tasks 1-9 (individual steps, dorsal), and the other half completed tasks 12-20 (individual steps, palmar).

Before each task, participants rinsed their hands to remove residual fluorescent concentrates. Then, participants were provided with 3 ml alcohol handrub on their left hand and asked to perform a task. Finally, photographs of participants’ hands were taken using an RGB camera both with and without UV light.

**Fig. 2:**
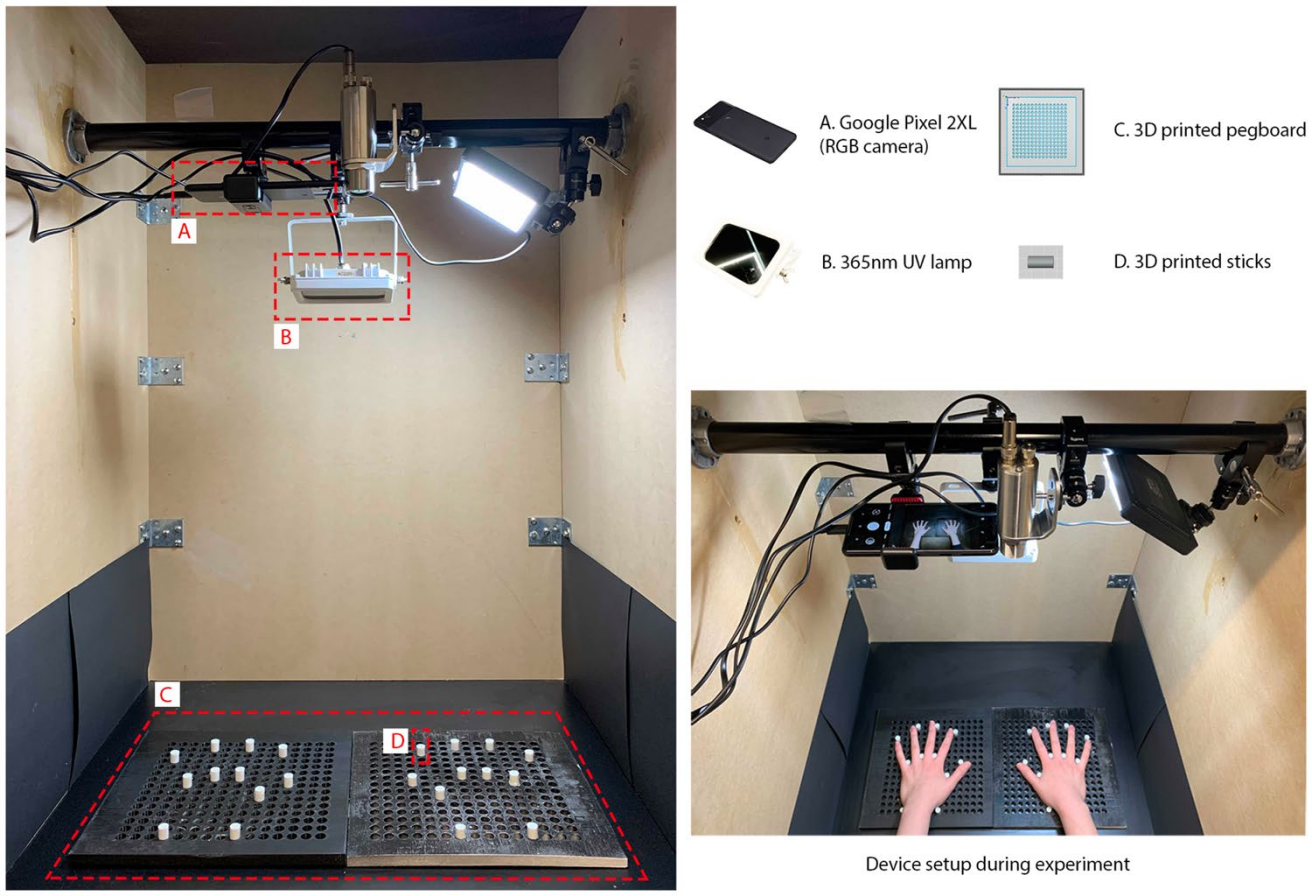
Experimental setup

### Hardware Setup

We built a darkened box to observe skin coverage with fluorescent handrub (details are shown in Fig. 2). To capture RGB images, we used an RGB camera from a smartphone (Pixel 2XL, Google LLC) with a resolution of $4032 \times 3024$ pixels, which was mounted centrally in the upper part of the box (Fig. 2A). We captured UV photographs using a 365 nm UV-A lamp with an effective irradiance of 30 $mW / cm^2$. The distance between the UV-A lamp and the observation board was set to 55 cm.

To minimize participants’ hand movements during the observation, and hence reduce the misalignment between images taken using the RGB camera with and without UV light, we 3D printed a pair of pegboards and sticks that helped participants keep their hands in the same position (Fig. 2C, D).

Regarding the fluorescent handrub, we mixed an antimicrobial alcohol gel (Microshield Angel Blue, Schülke & Mayr GmbH) with a fluorescent alcohol gel (Glitterbug Gel, OnSolution Pty Ltd) using a ratio of 3:2. This ratio was chosen in pilot tests, where we aimed to maximize the fluorescence of the mix under UV light, while maintaining desirable viscosity and thermal properties.

### Computational Analysis

**Fig. 3:**
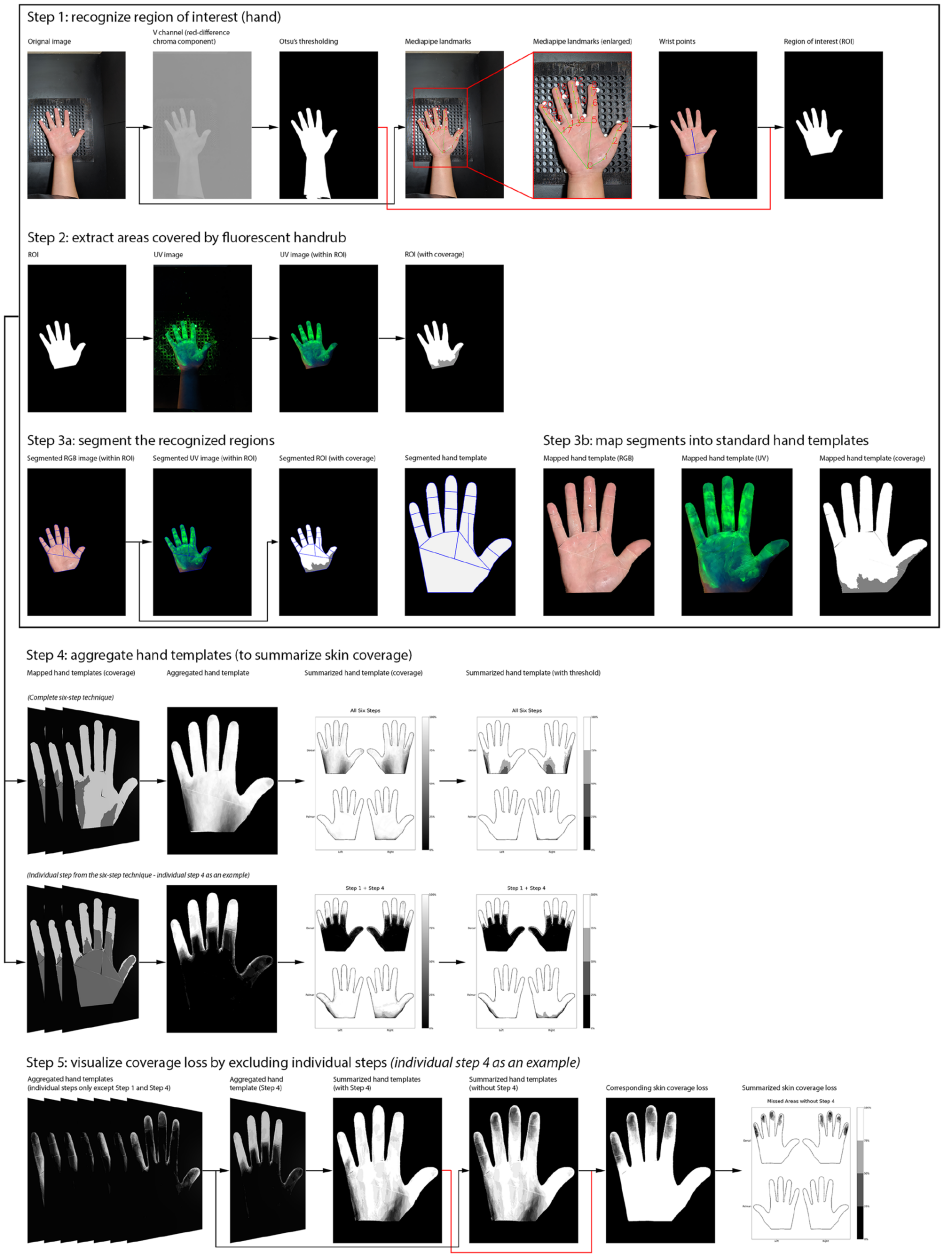
Our computational system. Steps 1 to 3 were repeated for each image collected from the experiment, and Steps 4 and 5 were repeated for each individual step in the six-step hand hygiene technique

The computational analysis involved five steps (details are shown in Fig. 3). The first step was to recognize the hand regions in the RGB images. We, therefore, transferred the RGB images taken under white light into the YUV color system. We then used the V channel (red-difference chroma component) and Otsu’s method for automatic image thresholding [14]. After finding the largest contours inside the binary images, the hands were segmented from the background. Since our study focused on hand hygiene, we further segmented regions above the wrists from the detected hand as Region of Interest (ROI). To recognize wrist points, we used MediaPipe to extract landmarks for each hand [15]. By connecting Point 0 and Point 9 (Fig. 3, Step 1, Subfig. 4), we then created a perpendicular line to intersect with the hand contour, and these points of intersection were considered as wrist points.

Next, we extracted the area covered by fluorescent handrub from the RGB images taken under the UV lamp. Since the fluorescent concentrate used in the experiment glowed in green under the UV lamp, we transferred these images to the HSB color system through OpenCV and used the H (Hue) channel to specifically detect areas within the green color range by using a threshold ($25 \le H \le 97$) [16]. To minimize the impact of residual fluorescent concentrates from previous tasks that are considered noise, we used the B (Brightness) channel to remove areas within the green color range but with low brightness ($0 \le B \le 60$).

The third step was to map the recognized hand regions into hand templates, but due to the diverse shapes, gestures, and positions of participants’ hands, the mapping procedure could not be achieved by simple transformations. Thus, we first split recognized hand regions and hand templates into 18 segments based on the landmarks generated by MediaPipe (and manual labels for the standard hand templates) and finger-web points (convexity defects of hand contours) calculated by OpenCV [16]. After that, the segments of the hand regions were matched and mapped to the corresponding segments of the standard hand templates, and the mapping procedure relied on the homography matrix calculated from the corners of each segment pair.

The fourth step was to aggregate the generated hand templates across all the applicable participants for each task (the aggregated hand templates are shown in Fig. 5). Before this step, a visual inspection was required to exclude inaccurate hand templates caused by hand movement (during the observation) and misalignment (caused by the algorithm). We then aggregated the generated hand templates which belonged to the same task and the same hand side. Each point within the aggregated hand templates represented the probability of being covered by fluorescent handrub after performing the corresponding task. There were two types of points removed from the aggregated hand templates for error correction. One type was points falling outside the boundaries of the standard drawing, while another was points within the hand templates but without coverage information. By aggregating hand templates from the same task, the skin coverage of the respective task was visualized. Because continuous grayscale values could be difficult to visually interpret, we further grouped each point into four categories, which were 0%-25%, 25%-50%, 50%-75%, and 75%-100% probability of coverage.

The last step was to visualize the estimated coverage loss by excluding individual steps from the six-step technique (the hand templates to visualize coverage loss are shown in Fig. 7). We first estimated the coverage of a combination of individual steps through hand templates from Step 4. Each point in an aggregated hand template represents the probability of areas covered after the individual step, but the aggregated hand templates from different tasks might overlap. Thus, the probability of a point from the hand template covered after a combination of individual steps was estimated as the maximum probability across all the included steps from the same hand template point. The procedure was repeated for all the points in the hand templates. Then, the two hand templates that accumulated coverage from all the individual steps with or without the individual step in question were subtracted to highlight the exclusive areas covered by the specific step. The estimated coverage loss by excluding individual steps was equal to the inverted subtraction results as $Probability\;(coverage\;loss) = 100\% - Probability\;(exclusive\;coverage)$. Finally, we applied the aforementioned probability thresholding (0%-25%, 25%-50%, 50%-75%, and 75%-100%) to facilitate visual interpretation. We noted that the results only considered the coverage loss due to excluding steps from the six-step technique and did not contain those areas that remain untreated when the complete six-step technique is used.

### Statistical Analysis

Our study focused on deriving both visual and quantitative assessments of skin coverage. For the complete six-step hand hygiene technique, the coverage percentage for different hands and sides was grouped by each participant and averaged. Pearson’s correlation was run to determine the relationship between the four different hand sides (*ie* dorsal left, dorsal right, palmar left, and palmar right).

For the individual steps of the six-step hand hygiene technique, we calculated the estimated coverage loss when we excluded one of the individual steps. We did this by dividing the size of the untreated areas caused by excluding an individual step by the corresponding hand areas (Fig. 7).

We presented discrete variables as numbers and percentages and continuous variables as mean (SD), while p values less than 0.05 were considered statistically significant. Statistical analyses was conducted using Python (version 3.6.8) and statsmodels (version 0.9.0).

## Results

Between March and April 2021, 32 participants were recruited and consented to the study. We discarded three participants’ data (Participants 1, 13, and 22) because of poor observance to the study protocol. Twenty-nine participants’ data were then processed, generating 754 mapped hand templates. Of these, 13 mapped hand templates were discarded due to misalignment. In total, 741 mapped hand templates were aggregated to visualize the skin coverage of the complete six-step hand hygiene technique or individual steps.

**Fig. 4:**
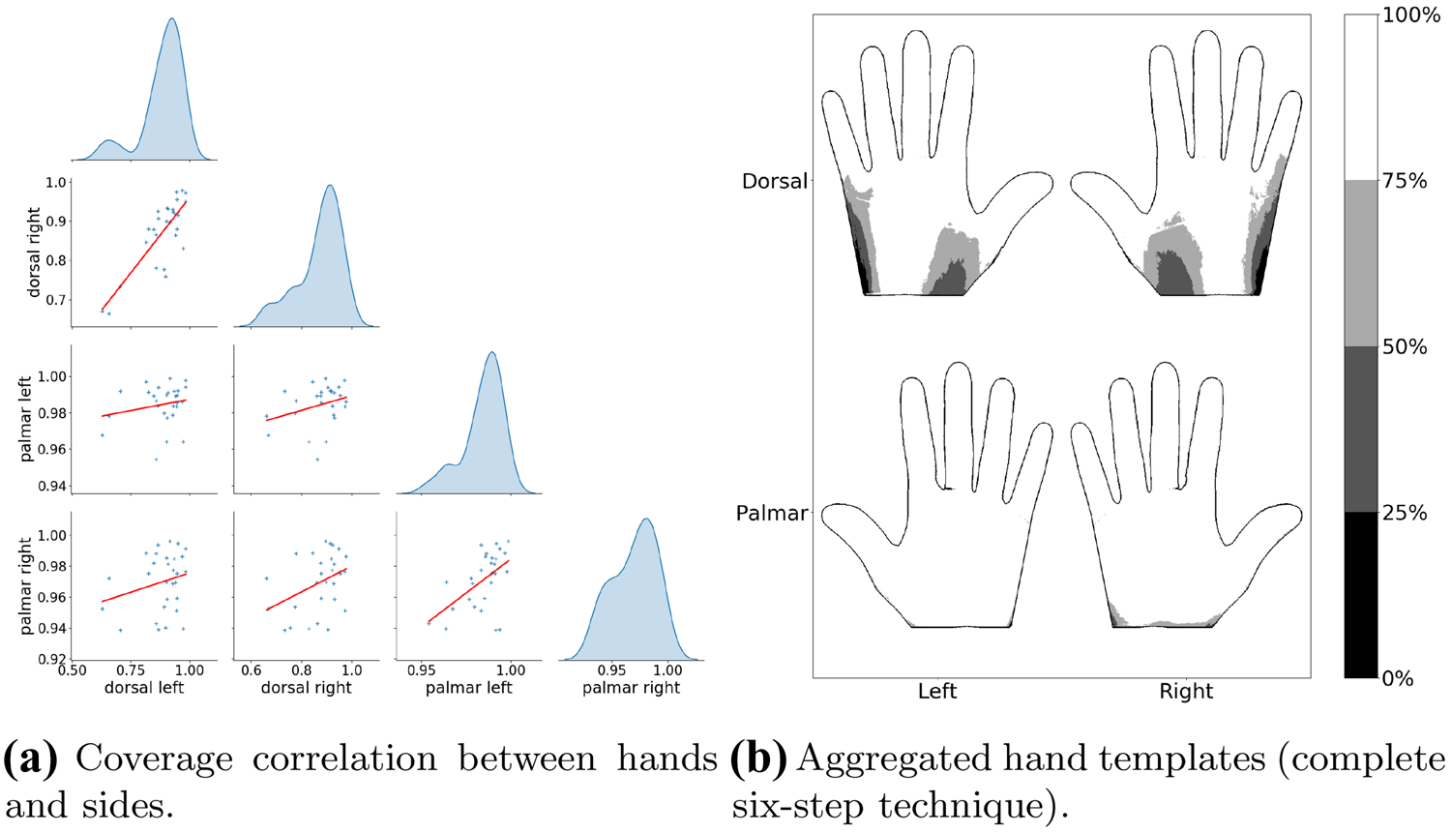
Using the complete six-step hand hygiene technique, we estimated the resulting hand skin coverage. **a** Individual participant performance; blue dots are participants, and red lines are regression lines. **b** Visual summary of achieved skin coverage. Each pixel within the hand template indicates the likelihood of being covered; darker color means the point is less likely to be covered

**Table 3:** Coverage loss when excluding an individual step from the six-step hand hygiene technique. The percentage is calculated by dividing the untreated areas caused by excluding an individual step into their corresponding hand areas (Fig. 7). Step 1 is skipped since it is a necessary prerequisite for other steps. We highlight potentially redundant steps in bold

**Step Hand side**	**dorsal left**	**dorsal right**	**palmar left**	**palmar right**
2R	39.5%	0.0%	0.0%	0.0%
2L	0.0%	40.4%	0.0%	0.0%
**3**	**0.0%**	**0.1%**	**0.1%**	**0.1%**
4	6.0%	6.3%	0.2%	0.2%
5R	3.4%	0.1%	1.6%	0.1%
5L	0.2%	4.8%	0.1%	0.7%
**6R**	**0.0%**	**0.0%**	**0.0%**	**0.1%**
**6L**	**0.0%**	**0.0%**	**0.1%**	**0.7%**

We have visualized each participant’s performance in Fig 4a. Pearson’s correlation was run to determine the relationship between the skin coverage for different hands and sides. There was a strong, positive correlation between dorsal left and dorsal right ($r = 0.8, n = 29, p < 0.001$) and a moderate, positive correlation between palmar left and palmar right ($r = 0.53, n = 29, p = 0.003$), while the remaining correlations were around or less than 0.3. The results showed that participants with comprehensive coverage on one hand side were likely to achieve satisfactory coverage on the same side of the other hand, but even when participants adequately cleaned their hands on one side, the opposite side might still be soiled.

We visualized the overall aggregate skin coverage achieved by all participants in Fig. 4b. The palmar side was nearly fully covered ($97.7\%, SD = 2.0\%$) by the alcohol-based handrub, while the untreated areas were mainly located on the dorsal side ($88.0\%, SD = 9.4\%$). More specifically, the skin areas around the abductor digiti minimi and the first dorsal interosseous were unlikely to be covered by the alcohol-based hand rub after performing the complete six-step technique.

**Fig. 5:**
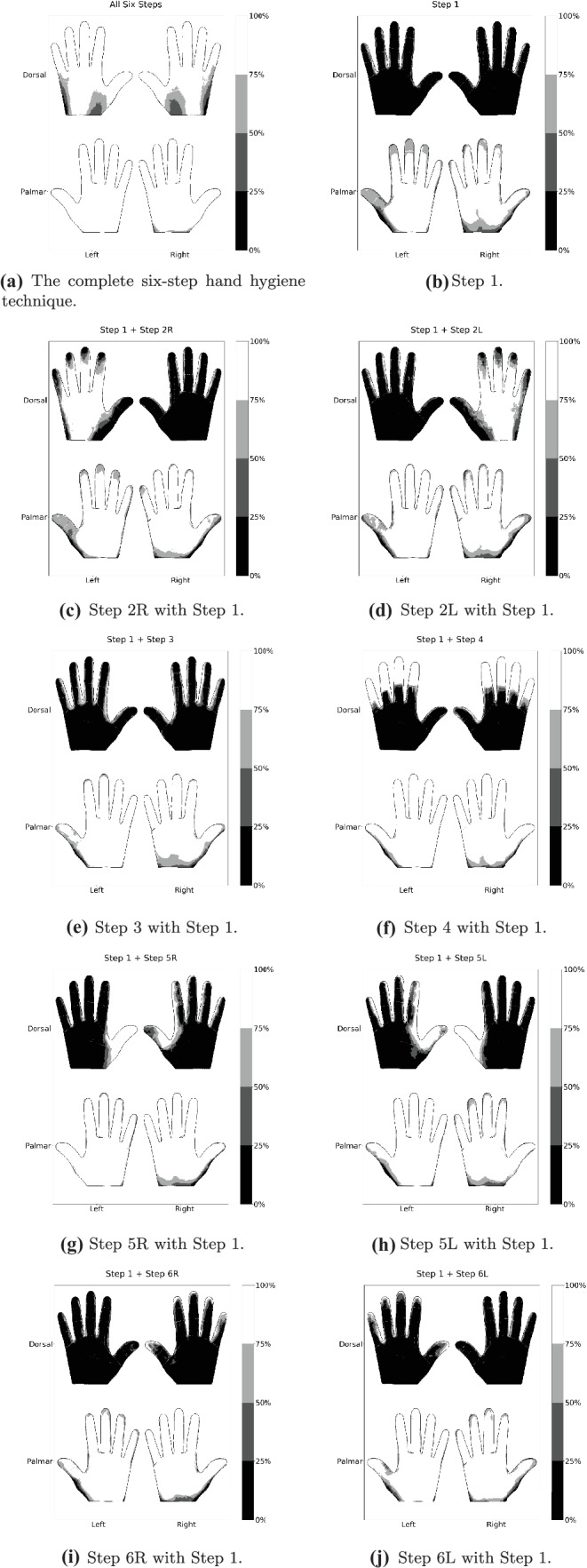
The skin coverage of the complete six-step hand hygiene technique, or each individual step. Each pixel within the hand template indicates the percentage of participants that covered it; darker color means the point is less likely to be covered

We estimated the coverage loss when excluding any individual step from the six-step hand hygiene technique. The numeric results can be seen in Table 3. We found that the coverage loss, when independently excluding Step 3, Step 6R, or Step 6L, was less than 1% for all hand sides. Moreover, the change when simultaneously excluding all three steps was also less than 1% for all hand sides: less than 0.1% for dorsal left and right, 0.3% for palmar left, and 1% for palmar right. The results indicated no significant coverage loss when excluding these steps, and thus, these steps might be potentially redundant in terms of skin coverage. In Fig. 5, we have visualized the skin coverage when completing any of the individual steps, and in Fig. 7, we have visualized the estimated coverage loss when excluding individual steps from the six-step hand hygiene technique.

**Fig. 6:**
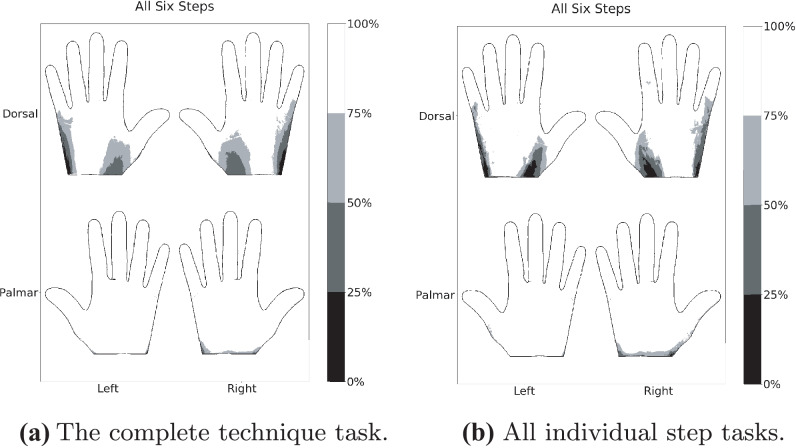
The skin coverage of complete technique task, or accumulated skin coverage of all individual step tasks. Each pixel within the hand template indicates the percentage of participants that covered it; darker color means the point is less likely to be covered

**Fig. 7:**
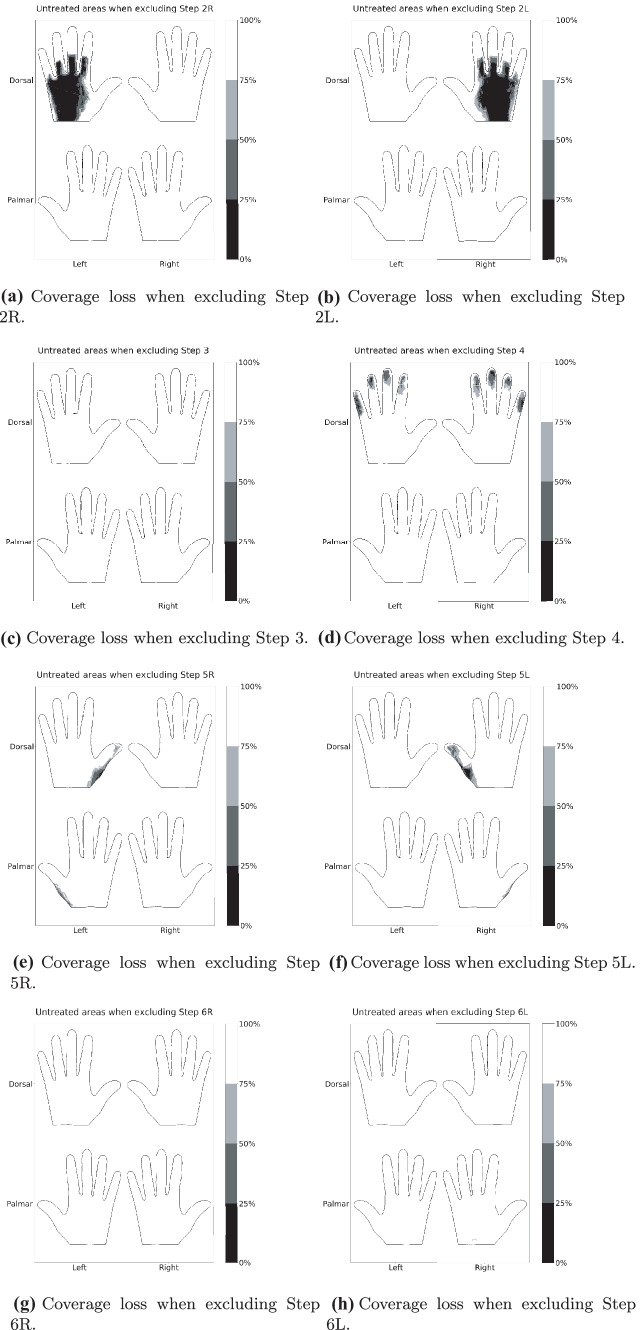
The coverage loss when excluding individual steps from the six-step hand hygiene technique. Each pixel within the hand template indicates the chance that it will be untreated; darker color means the pixel is more likely to be untreated

Finally, to validate our experimental design and choice of tasks, we compared the hand templates that visualize accumulated coverage of all individual steps vs. the hand templates generated from conducting the complete technique. The results, shown in Fig. 6, showed high similarity, suggesting that breaking down the procedure in individual steps did not compromise the validity of the results.

## Discussion

We presented an automated system to assess the effectiveness of a hand hygiene technique in terms of skin coverage with fluorescent handrub. The system was first used to precisely measure the skin coverage of the complete six-step hand hygiene technique and separately for the individual steps. By further aggregating the coverage outcomes into standard hand templates, we were also able to highlight the frequently untreated areas across many participants, and to identify potentially redundant steps.

The frequently untreated areas were mainly located at the dorsal side around the abductor digiti minimi and the first dorsal interosseous, since no step of the six-step hand hygiene technique might be specifically designed to cover these areas. Our findings agree with previous results [6, 7] One potential way to reduce the untreated areas could be introducing a new step complementing the existing six-step technique. For example, the new step could be rotational rubbing of each wrist clasped in the opposite hand, which has already been widely adopted in clinical practices [17].

Our work has also highlighted the estimated coverage loss when excluding individual steps from the six-step hand hygiene technique. This allowed us to identify Steps 3, 6R, and 6L as potentially redundant in terms of skin coverage, since other steps already sufficiently cover their targeted areas. For example, Step 3 was designed to cover interdigital webs of the palms, while Steps 1 and 2 covered interdigital webs of both sides of the hands. Also, given the strong correlation between the visual evaluation of UV tests and the degree of bacterial reduction, these three steps might not be associated with superior microbiological efficacy [11, 12]. Moreover, since HCWs’ adherence to the whole six-step hand hygiene technique is low and each individual step takes approximately three seconds to complete, simplifying the six-step hand hygiene technique by removing redundant steps might potentially result in an increase of HCWs’ hand hygiene compliance and adherence [8, 18].

This study has some limitations. First, this study measured hand hygiene quality in terms of skin coverage with fluorescent handrub without further microbiological validation, but a strong correlation between the visual evaluation of UV tests and the degree of bacterial reduction has previously been reported [11, 12]. Second, this study was conducted with participants who were not familiar with the formal hand hygiene techniques, to assess the practicality and effectiveness of the six-step hand hygiene technique, and thus, results may differ for people who received formal hand hygiene training. Third, since the RGB camera was mounted centrally in the upper part of the darkened box, the sides of fingers and palms may be indistinct in the images; thus, future work could involve more cameras to further minimize the blind spots and provide a more comprehensive view. Fourth, residual fluorescent concentrates often occurred during the experiment when participants did not properly wash off the fluorescent concentrates from their hands after completing the tasks. Thus, we asked participants to rewash their hands if the amount of residual fluorescent concentrate was substantial, while we also set a threshold to filter residual UV concentrates computationally. In addition, the computational analysis algorithm may result in misalignment when mapping coverage information to standard hand templates. To prevent this misalignment from impacting our results, we manually removed the misaligned hand templates and filtered out points that were outside the standard hand templates. Future work is needed to further improve the computational analysis algorithm.

## Conclusions

We presented a system to computationally assess the effectiveness of a hand hygiene techniques. This study used our system to evaluate the six-step hand hygiene technique, highlight the resulting frequently untreated areas, and identify three redundant steps.
